# One of these strains is not like the others: *C. elegans* DW102 has an altered dependence on *brc-1* and *brd-1* for regulation of *cyp* gene transcription

**DOI:** 10.17912/micropub.biology.001152

**Published:** 2024-06-04

**Authors:** Ishor Thapa, Marlo K. Sellin Jeffries, Mikaela D. Stewart

**Affiliations:** 1 Biology Department, Texas Christian University, Fort Worth, Texas, United States

## Abstract

Several strains of *Caenorhabditis elegans* with mutations in *brc-1* or *brd-1* are readily available to aid in elucidating the functions of these two genes in DNA damage repair, meiosis, and gene repression. DW102 is the only *C. elegans* strain to our knowledge with mutations in both *brc-1* and *brd-1* . However, several groups have reported the DW102 strain is indistinguishable from wild-type when observing levels of embryonic lethality, sensitivity to radiation, and rates of male progeny, while strains with mutations in either *brc-1* or *brd-1* display increased occurrence of these phenotypes. Here, RT-qPCR analysis of the *cyp-13A* gene family, reveals distinctive and aberrant expression patterns in DW102 compared to other *brc-1* or *brd-1* mutant strains underscoring the need for caution in choosing this strain to draw conclusions about *brc-1* and *brd-1* functions.

**Figure 1. Cyp-13A gene expression analysis in WT and DW102 C. elegans.**
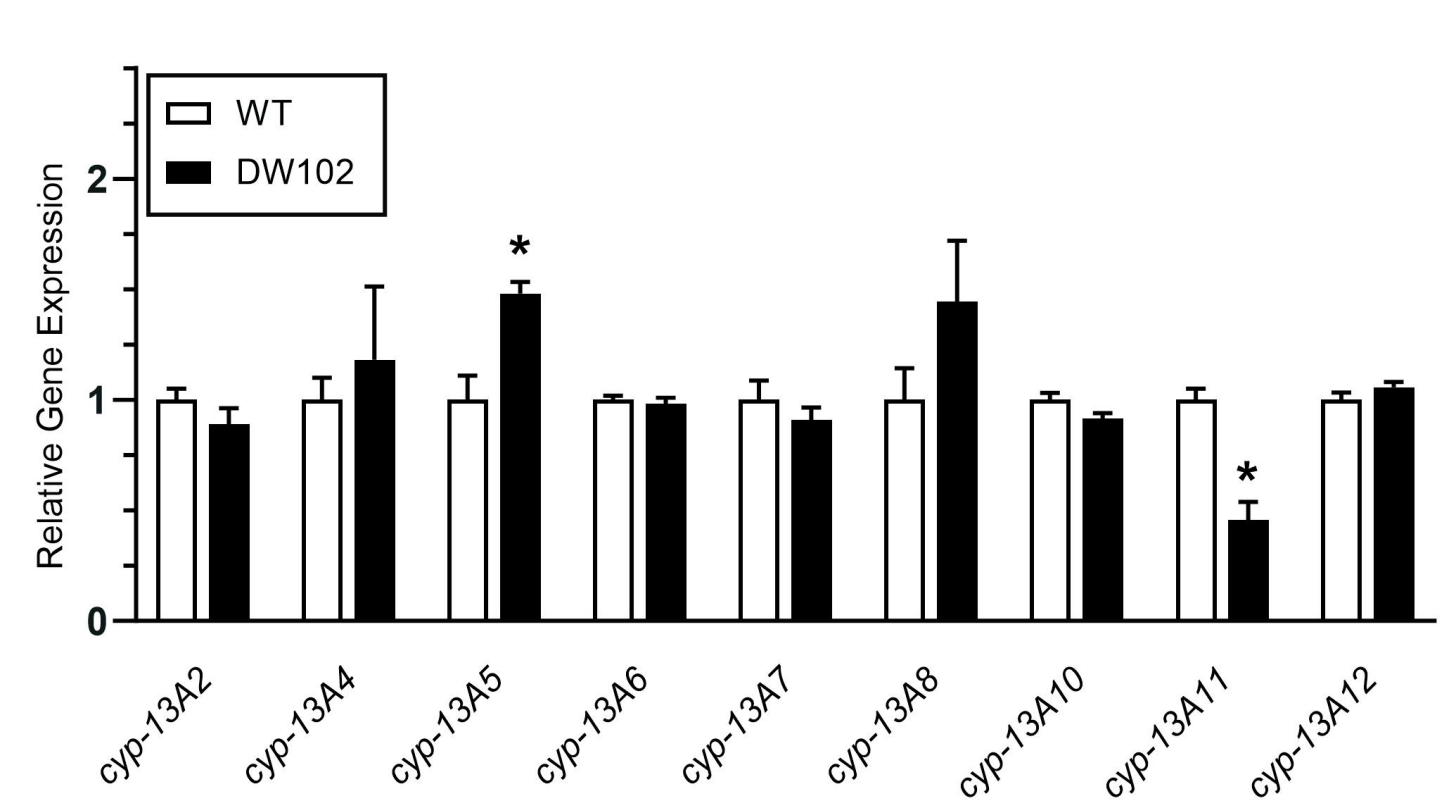
The expression profiles of *cyp-13A2* , *cyp-13A4* , *cyp-13A5* , *cyp-13A6* , *cyp-13A7* , *cyp-13A8* , *cyp-13A10* , *cyp-13A11* , and *cyp-13A12* in wild-type N2 (WT) and DW102 strains were assessed using RT-qPCR and normalized to the expression of the reference gene, *tba-1* . Data are presented as the mean starting quantity relative to WT. Error bars represent the standard error of the mean (± SEM) for four biological replicates. The deviation from WT was determined using student's t-test with * indicating a statistically significant value (p-value < 0.05).

## Description

We recently reported repression of *cyp* genes as a conserved function shared by the human tumor-suppressor genes *BRCA1* and *BARD1* and the *C. elegans* homologs *brc-1* and *brd-1* (Thapa et al., 2023) . Proteins produced by these genes, BRC-1 and BRD-1 , form a heterodimer that allows for ubiquitylation of nucleosomes on histone H2A. In human cells, ubiquitylated H2A is the signal for *cyp* gene repression, and loss of either *BRCA1* or *BARD1* leads to loss of *cyp* gene repression (Savage et al., 2014; Stewart et al., 2018) . Likewise, in *C. elegans* we reported loss of repression of *cyp-13A11* and *cyp-13A5* in a CRISPR-generated *brc-1* deletion allele ( *gk5332* ) and in a *brd-1* mutant allele ( *dw1* ). However, mutation of *brc-1* led to loss of repression of additional *cyp* genes ( *cyp-13A2* and *cyp-13A10* ) that are not affected by the *brd-1* mutation, while *brd-1* mutation led to loss of repression of other *cyp* genes ( *cyp-13A4 , cyp-13A6 , cyp-13A8* , and *cyp-13A12* ) not observed for the *brc-1* mutant.

To determine if different profiles for the two strains are due to independent functions of BRC-1 and BRD-1 , we measured expression of all detectable *cyp-13A* genes ( *cyp-13A2* , *cyp-13A4* , *cyp-13A5* , *cyp-13A6* , *cyp-13A7* , *cyp-13A8* , *cyp-13A10* , *cyp-13A11* , and *cyp-13A12* ) in the DW102 strain using RT-qPCR. This DW102 strain was originally described as a *brc-1* mutant strain and is commonly referred to as the *brc-1 ( tm1145 )* allele, but has been used in recent years as a double *brc-1 / brd-1* mutant after *brd-1* genotyping revealed mutation of both genes (Janisiw et al., 2018) . The expectation is that mutation of both genes would result in loss of repression of all eight *cyp* genes impacted by either *brc-1* or *brd-1* . Contrary to expectation, we only observed increased expression of one of the expected genes, *cyp-13A5* ( Figure 1 ) *.* Surprisingly, we report the opposite effect in expression of *cyp-13A11* ; while loss of *brc-1* or *brd-1* function led to increases in expression (Thapa et al., 2023) , here we observed decreased expression in DW102 compared to wild-type (WT). BRC-1 and BRD-1 are predicted to mediate gene repression through H2A ubiquitylation, so the continued repression of many *cyp-13A* genes in the DW102 strain is particularly surprising in light of the finding that DW102 *C. elegans* demonstrate loss of ubiquitylated H2A in proximity to satellite repeats in a comparable fashion to *C. elegans* with the *brc-1 ( ok1261 )* allele (Padeken et al., 2019) . It is important to note that ubiquitylated H2A levels were measured in L1 larval stage, while the expression patterns reported here are in a later larval stage, L4.

Several other *brc-1* and *brd-1* phenotypes differ between the widely-used DW102 strain and other *brc-1* or *brd-1* mutant strains. The embryonic lethality, production of male progeny, and sensitivity to irradiation is similar for DW102 and WT, while another *brc-1* mutant allele ( *xoe4* ) and *brd-1* mutant alleles ( *ok1623* and *dw1* ) show significant increases in these three phenotypes compared to WT (Li et al., 2018) . Furthermore, CRISPR-generated *brc-1* null mutant strains are unable to load exogenous BRD-1 onto DNA during meiosis, while the DW102 strain does not demonstrate this defect (Janisiw et al., 2018) . More recently, Hariri et al. (2023) reported enhanced embryonic lethality in double mutant *C. elegans* carrying a *hsr-9* mutation along with the *brc-1 ( xoe4 )* or *brd-1 ( xoe18 )* allele, but DW102 animals with a *hsr-9* mutation have rates of embryonic lethality that are indistinguishable from wild type. While some of the observed affects could be explained by DW102 retaining some BRC-1 functions (the resulting BRC-1 protein in DW102 is missing 71 amino acids from a domain of unknown function) (Janisiw et al., 2018) , it is a challenge to envision how this hypothesis explains the WT-like phenotypes in the absence of *brd-1* and the observed decrease in *cyp-13A11* expression compared to WT. Given that *brc-1* and *brd-1* are separated by many genes on chromosome III and both *brc-1* and *brd-1* contain mutations in DW102 , a more likely hypothesis is that this strain contains additional gene mutations that are unreported. We urge caution when interpreting BRC-1 and BRD-1 functions based on data generated from the DW102 strain and suggest parallel use of additional *brc-1* and *brd-1* mutant strains if DW102 must be utilized in future experiments.

## Methods

***C. elegans* strains and maintenance** : Worm WT ( N2 Bristol) was generously provided by Dr. Phil Hartman (Texas Christian University, Texas, USA). The strain of DW102 [ *brc-1 ( tm1145 )* ] was acquired from Caenorhabditis Genetics Center, University of Minnesota (funded by NIH Office of Research Infrastructure Programs P40 OD010440). All *C. elegans* were maintained at 20°C on nematode growth media (NGM) seeded with *E. coli* OP50 . A synchronized population of L1 *C. elegans* was grown on *E. coli* -seeded NGM plates to obtain L4-stage synchronization. A pool of 2000 L4 worms per biological replicate was flash-frozen using dry ice and stored at -80°C until RNA isolation. Four biological replicates were processed for each strain.

**RNA isolation and RT-qPCR:** RNA isolation and RT-qPCR procedures were followed as previously described (Thapa et al., 2023) . Briefly, for each biological replicate, total RNA was extracted from a pool of 2000 flash frozen L4 worms using the Maxwell 16 LEV simplyRNA Tissue kit. Total RNA was quantified and assessed for purity via spectrophotometric analysis carried out by the NanoDrop 1000 (ThermoFisher Scientific), and all samples were confirmed to have 260/280 absorbance ratios > 2.0. First-strand DNA synthesis reactions, containing 2 mL of qScript cDNA Supermix (Quantabio) and 8 mL of 50 ng/mL RNA diluted into nuclease-free water, were carried out using a TC100 thermal cycler (BioRad) with a program of 5 min at 25°C followed by 30 min at 42°C and 5 min at 85°C. Each qPCR reaction consisted of 0.4 mL of cDNA, 4.3 mL of nuclease-free water, 0.3 mL of primer mix, and 5 mL of SYBR Green FastMix (Quantabio) with an activation step at 95°C for 30 s, followed by 40 cycles of denaturation at 95°C for 10 s and annealing for 15 s at the primer-specific temperatures reported previously (Thapa et al., 2023) . The optimal cycle threshold was set automatically by the CFX software program. Standard curves were generated from serially-diluted standards ran on each plate. The equation of the best-fit line describing the relationship between the starting quantity (SQ) of cDNA standards and the Ct values obtained were used to calculate SQ of the samples from their Ct values and estimate the efficiency of primers (80-97%). A melt curve analysis was carried out for each run to ensure the presence of a single qPCR product. All samples were run in triplicate and any replicate showing a deviation exceeding two standard deviations from the mean for a specific gene was regarded as an outlier and excluded prior to analysis. A stable reference gene, *tba-1* , was used to normalize the gene expression data by presenting each sample as the mean *cyp* SQ relative to the mean *tba1* SQ. The average SQ of *tba-1* does not vary across strains. Data were analyzed and visualized using GraphPad Prism version 10.0. To enhance data visualization, gene expression data were normalized to the mean expression of the WT group and presented accordingly. To test for significant differences in gene expression between the DW102 and WT ( N2 ) groups, a student's t-test was performed and alpha was set to 0.05.
